# A Novel m1A-Score Model Correlated With the Immune Microenvironment Predicts Prognosis in Hepatocellular Carcinoma

**DOI:** 10.3389/fimmu.2022.805967

**Published:** 2022-03-24

**Authors:** Mingxing Zhao, Shen Shen, Chen Xue

**Affiliations:** ^1^ Department of Traditional Chinese Medicine, The First Affiliated Hospital of Zhengzhou University, Zhengzhou, China; ^2^ Gene Hospital of Henan Province, Precision Medicine Center, The First Affiliated Hospital of Zhengzhou University, Zhengzhou, China; ^3^ Department of Infectious Diseases, The First Affiliated Hospital of Zhengzhou University, Zhengzhou, China

**Keywords:** HCC, m1A modification, tumor microenvironment, prognosis, biomarker

## Abstract

RNA methylation plays crucial roles in gene expression and has been indicated to be involved in tumorigenesis, while it is still unclear whether m1A modifications have potential roles in the prognosis of hepatocellular carcinoma (HCC). In this study, we comprehensively analyzed RNA sequencing (RNA-seq) data and clinical information using The Cancer Genome Atlas (TCGA) and Gene Expression Omnibus (GEO) databases. We collected 10 m1A regulators and performed consensus clustering to determine m1A modification patterns in HCC. The CIBERSORT method was utilized to evaluate the level of immune cell infiltration. Principal component analysis was used to construct the m1A-score model. In the TCGA-LIHC cohort, the expression of all 10 m1A regulators was higher in tumor tissues than in normal control tissues, and 8 of 10 genes were closely related to the prognosis of HCC patients. Two distinct m1A methylation modification patterns (Clusters C1 and C2) were identified by the 10 regulators and were associated with different overall survival, TNM stage and tumor microenvironment (TME) characteristics. Based on the differentially expressed genes (DEGs) between C1 and C2, we identified three gene clusters (Clusters A, B and C). C1 with a better prognosis was mainly distributed in Cluster C, while Cluster A contained the fewest samples of C1. An m1A-score model was constructed using five m1A regulators related to prognosis. Patients with higher m1A scores showed a poorer prognosis than those with lower scores in the TCGA-LIHC and GSE14520 datasets. In conclusions, our study showed the vital role of m1A modification in the TME and progression of HCC. Quantitative evaluation of the m1A modification patterns of individual patients facilitates the development of more effective biomarkers for predicting the prognosis of patients with HCC.

## Introduction

Specific chemical modifications, such as modifications of DNA and proteins, are classical ways of regulating molecular function. Various regulators responsible for regulating protein and DNA modifications are potential targets of cancer treatment (1). Compared with protein and DNA modifications, RNA modification is a new frontier of this area (1). Recently, with the advancement of next-generation sequencing technologies, RNA modifications have gained much attention because they participate in several physiological and pathological processes. To date, more than 150 RNA modifications have been identified (2, 3). Among them, RNA methylation is the most extensively studied type. Many studies have indicated that RNA methylation plays an essential role in diverse physiological processes in human cancers (4). RNA methylation plays crucial roles in gene expression and has been proven to be involved in tumorigenesis by regulating cancer cell proliferation, invasion, metastasis, and drug resistance.

N^1^-methyladenosine (m1A) is an important posttranscriptional RNA modification that was first discovered in tRNA in 1966 (5). Decades later, m1A was found in tRNA (5), rRNA (6, 7), mitochondrial RNA (8, 9) and mRNA (10). Under the action of a methyltransferase, m1A can be formed by adding a methyl group at the N1 position of adenosine, which blocks Watson-Crick base pairing, affecting transcription and protein-RNA interactions (11). m1A is a dynamic and reversible RNA modification that is mediated by RNA-modifying proteins called “writers” (methyltransferases) catalysing the formation of methylation, “readers” (modified RNA-binding proteins) reading the information of methylation modification, and “erasers” (demethylases) detecting the methylation modification of RNA. Emerging data suggest that m1A regulators play important roles in tumorigenesis and progression. Silencing TRMT10C has been found to inhibit the malignant characteristics of ovarian cancer and cervical cancer tumor cells (12). The writer TRMT6 was reported to be significantly upregulated in hepatocellular carcinoma (HCC) tissues compared to adjacent tissues, and higher expression of TRMT6 was correlated with poor prognosis in HCC patients (13). The eraser ALKBH3 contributes to lung cancer development and correlates with recurrence-free survival in adenocarcinoma (14). However, previous studies have mainly focused on only one m1A regulator in cancer. Therefore, comprehensive analysis of the genetic variations in these m1A regulators and their relationships with the tumor microenvironment (TME) and clinical characteristics will enhance our understanding of the molecular mechanisms of m1A in cancer.

Here, we integrated the gene expression information and clinical data of HCC patients to comprehensively evaluate the genetic variations in 10 m1A regulators, m1A modification patterns, and the association between m1A modification patterns and TME characteristics. Additionally, we generated an m1A model based on m1A regulators and related genes that can quantify the m1A modification pattern in individual patients. The m1A score is closely associated with tumor immune microenvironment (TIME) characterization and displays potential in predicting the prognosis of patients with HCC.

## Methods

### Data Source and Preprocessing

RNA sequencing (RNA-seq) data and clinical information were downloaded from the Gene Expression Omnibus (GEO) and The Cancer Genome Atlas (TCGA) databases. mRNA, single nucleotide variation (SNV), copy number variation (CNV) and clinical data were obtained from the TCGA database (https://portal.gdc.cancer.gov/). The GSE14520 cohort from GEO (https://www.earthobservations.org) was included for further analysis. Patients without complete survival data were excluded from further analysis.

### CIBERSORT

CIBERSORT is a machine learning approach for characterizing the cell composition of a tumor biopsy from gene expression data (http://cibersort.stanford.edu) and is a useful method for the high-throughput characterization of various cell types, such as tumor-infiltrating leukocytes (15). Usually, a feature matrix containing 22 functionally defined human immune subgroups (LM22) is used for verification. Here, we used the CIBERSORT method to calculate the proportions of 22 immune cells in the two m1A clusters.

### Construction and Validation of a Prognostic Risk Model

First, we identified the differentially expressed genes (DEGs) between distinct m1A clusters. The survival data and DEGs were analyzed by univariate Cox regression analysis. Second, least absolute shrinkage and selection operator (LASSO) Cox regression analysis was utilized to further narrow down the DEGs associated with prognosis. Finally, we performed multivariate Cox regression analysis to establish the prognostic model. The risk score was calculated by summing the risk coefficient of each gene.

### Generation of the m1A-Score Model

To quantify the m1A modification patterns of individual patients with HCC, we generated a scoring system named the m1A-score model. The procedures for establishing the m1A-score model were as follows. First, we performed principal component analysis to construct the m1A-score model. Principal components 1 and 2 were used as signature scores for each sample. The calculation formula was as follows: m1A-score=ΣPC1i+PC2i where i is the expression of the 5 genes (PON1, CFHR3, CAD, NT5DC2 and CDC20) that were screened from the prognostic risk model and related to the m1A clusters.

### Single-Sample Gene Set Enrichment Analysis (ssGSEA)

The ssGSEA method is used to calculate an enrichment score that represents the degree of absolute enrichment of a gene set (16). In this study, we performed gene set variation analysis (GSVA) using the GSVA (17) R package and the c2.cp.kegg.v7.0.symbols.gmt gene set. In addition, we used the limma package to perform differential analysis on the results of GSVA. The Pheatmap package was used to draw a heatmap.

### Cell Culture

The normal human liver cell line L02 and human liver cancer cell line HepG2 were obtained from the Chinese Academy of Sciences (Shanghai, China). The cell lines were maintained in Dulbecco’s modified Eagle’s medium (DMEM, Gibco USA) supplemented with 10% foetal bovine serum and 1% penicillin. All the cells were cultured at 37°C in a humidified incubator with a 5% CO2 atmosphere.

### Quantitative Reverse-Transcription PCR (qRT-PCR)

Total RNA was isolated from the L02 and HepG2 cell lines using TRIzol reagent (Invitrogen) and then reverse transcription was performed using the PrimeScript RT-PCR Kit (Takara, Japan) according to the manufacturer’s instructions. Relative mRNA levels were detected by an ABI7500fast PCR instrument. GAPDH was used as the internal control. The relative expression levels of the m1A-related regulator genes were normalized to the expression of GAPDH, which was calculated using the 2^-ΔΔCt^ method.

### Statistical Analysis

The statistical analyses in this study were carried out with SPSS 25 (IBM Corporation, Armonk NY) and R software (version 3.5.1). Student’s t-test was utilized to estimate the differences between two groups. For comparisons of more than two groups, one-way analysis of variance was used. Kaplan–Meier survival analysis and the log-rank test were used to establish survival curves and compare the differences. All P values were two-sided, and P < 0.05 was considered statistically significant.

## Results

### Features of Genetic Variations in m1A Regulators in HCC

A total of 10 m1A RNA modification regulators (including TRMT10C, TRMT61B, TRMT6/61A, YTHDF1, YTHDF2, YTHDF3, YTHDC1, ALKBH1, and ALKBH3) were included in the current study based on the findings of previously published studies (9, 18, 19). To describe the landscape of genetic alterations in m1A regulators in HCC, we assessed the degree of CNVs of individual m1A regulators. According to the degree of CNVs, CNVs were divided into three types: amplification, diploid and deletion. The proportions of the 10 genes with amplifications and deletions are shown in **Table 1**. According to the types of CNVs of individual regulators, we further explored the correlation between the expression of each regulator and CNVs, and the results are shown in **Figure S1A**. Additionally, in the 364 TCGA-liver hepatocellular carcinoma (LIHC) samples, most SNV mutations occurred in the TRMT10C, YTHDF1 and YTHDC1 genes (**Figure S1B**). All these results suggested that the genetic variations in m1A regulators might lead to expression and functional changes in those regulators that play a critical role in the occurrence, progression and prognosis of HCC.

**Table 1:** The proportion of 10 genes related to m1a modification that have undergone amplification and deletion.

		diploid	deletion	CNV-sum	amplification %	Deletion%
**writer**	TRMT10C	719	14	768	4.557291667	1.822916667
	TRMT61B	717	9	767	5.345501956	1.173402868
	TRMT6	657	25	773	11.77231565	3.234152652
	TRMT61A	645	108	768	1.953125	14.0625
**reader**	YTHDF1	665	100	768	0.390625	13.02083333
	YTHDF2	644	115	769	1.300390117	14.95448635
	YTHDF3	563	22	769	23.92717815	2.860858257
	YTHDC1	640	117	768	1.432291667	15.234375
**eraser**	ALKBH1	653	101	770	2.077922078	13.11688312
	ALKBH3	721	31	777	3.217503218	3.98970399

### Changes in m1A Regulators Were Correlated With the Prognosis of HCC Patients

**Figure 1:**
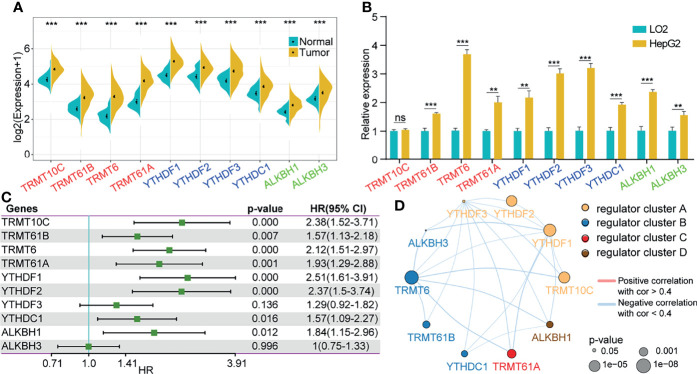
Changes in m1A regulators were correlated with the prognosis of HCC patients. **(A)** Expression of 10 m1A-related genes in tumor tissues compared to normal liver tissues from the TCGA-LIHC cohort. **(B)** Expression of 10 m1A-related genes in the HepG2 cell line compared with the normal cell line L02. **(C)** Univariate cox analysis of the 10 regulators. **(D)** Construction of the m1A regulatory network. **P < 0.01, ***P < 0.001; ns, nonsense.

Among the 10 m1A regulators, there were 4 modification writers, 4 readers and 2 erasers. We explored the expression of the 10 m1A regulators in tumor tissues compared with normal control tissues from the TCGA-LIHC cohort. We discovered that all regulators were more highly expressed in tumor tissues than in normal tissues (**Figure 1A**). Consistent with the expression in the TCGA-LIHC cohort, a total of 9 genes (Trmt61B, TRMT6, TRMT61A, YTHDF1, YTHDF2, YTHDF3, YTHDC1, ALKBH1, and ALKBH3) were highly expressed in the HepG2 cell line compared with the normal liver cell line L02. The expression level of TRMT10C was not significantly different between the HepG2 and L02 cell lines (**Figure 1B**). Furthermore, we performed univariate Cox analysis on the 10 genes by extracting clinical information from TCGA-LIHC, and the results showed that 8 of the 10 genes were closely related to the prognosis of HCC patients (**Figure 1C**). To comprehensively explain the associations among the 10 m1A regulators, we constructed a regulatory network by unsupervised cluster analysis to describe the interactions of m1A regulators and their influence on the prognosis of LIHC patients. These regulator genes were classified into 4 distinct clusters, as illustrated in **Figure 1D**.

### m1A Modification Patterns Mediated by 10 Regulators

**Figure 2:**
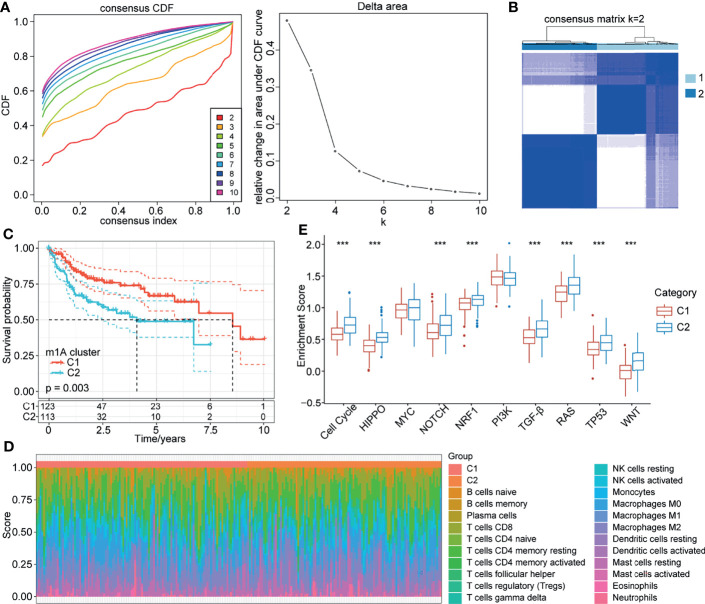
m1A modification patterns mediated by 10 regulators. **(A)** CDF curve and CDF delta area curve in TCGA-LIHC cohort. **(B)** Clustering heatmap when consensus k=2. **(C)** Kaplan-Meier curve of the prognostic relationship between the two clusters in the TCGA-LIHC cohort. **(D)** Proportions of 22 immune cell components in samples in C1 and C2. **(E)** Differences in the scores of 10 pathways related to tumor abnormalities in C1 and C2. ***P < 0.001.

We classified patients with different m1A modification patterns based on the expression of the 10 m1A regulators using the ConsensusClusterPlus R package. We set the parameters clusterAlg = “pam” and distance = “euclidean” to determine the optimal number of clusters according to the cumulative distribution function (CDF) and observe the CDF delta from the area curve. When the cluster number was 2, there was a relatively stable clustering result (**Figure 2A**); thus, we choose k=2 to obtain two distinct m1A clusters (C1 and C2) (**Figure 2B**). Further analysis of the prognostic characteristics of these two subtypes showed that there were prognostic differences between them, and C1 tended to have a prominent survival advantage (**Figure 2C**).

To further explore the biological behaviours of the m1A modification phenotypes, we focused on the TME cell infiltration characteristics of different m1A modification patterns. We used the CIBERSORT method to calculate the proportions of 22 immune cells for the two subtypes (**Figure 2D**). We also compared the score differences of 10 pathways related to tumor abnormalities in the different subtypes. The results showed that C2 patients with a poor prognosis had a higher enrichment score in the 8 pathways of the cell cycle, HIPPO, NOTCH, NRF1, TGF-beta, RAS, TP53 and WNT than C1 patients (**Figure 2E**). Additionally, we calculated the enrichment scores of 187 pathways for each sample by ssGSEA. The results showed that C1 was enriched in pathways mainly related to metabolism, and C2 was enriched in 18 pathways mainly related to the cell cycle and tumors (**Figure S2**).

### Association of m1A Modification Patterns With Clinical Characteristics of HCC Patients

**Figure 3:**
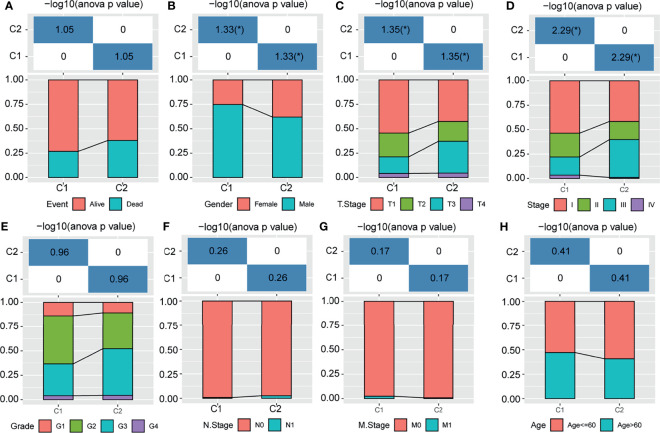
Distribution of m1A-related subtypes in clinical features. **(A–H)** The survival status, sex, T stage, stage, grade, N stage, M stage, and age of the m1A-modified subtypes. *P < 0.05.

To reveal the role of m1A modification patterns in the progression of HCC, we compared the various clinical characteristics of distinct patterns. We found that there was no difference in the survival status of patients with modified m1A patterns (**Figure 3A**, P>0.05). In terms of sex, T stage and stage, there were significant differences in the m1A-modified subtypes (**Figures 3B**–**D**, all P<0.05). There was no difference in the grade, N stage, M stage, or age of the m1A-modified subtypes (**Figures 3E**–**H**, all P>0.05). This finding suggests that m1A modification patterns might be markedly related to the prognosis and progression of HCC.

### Identification of Gene Clusters Related to m1A Modification Patterns

**Figure 4:**
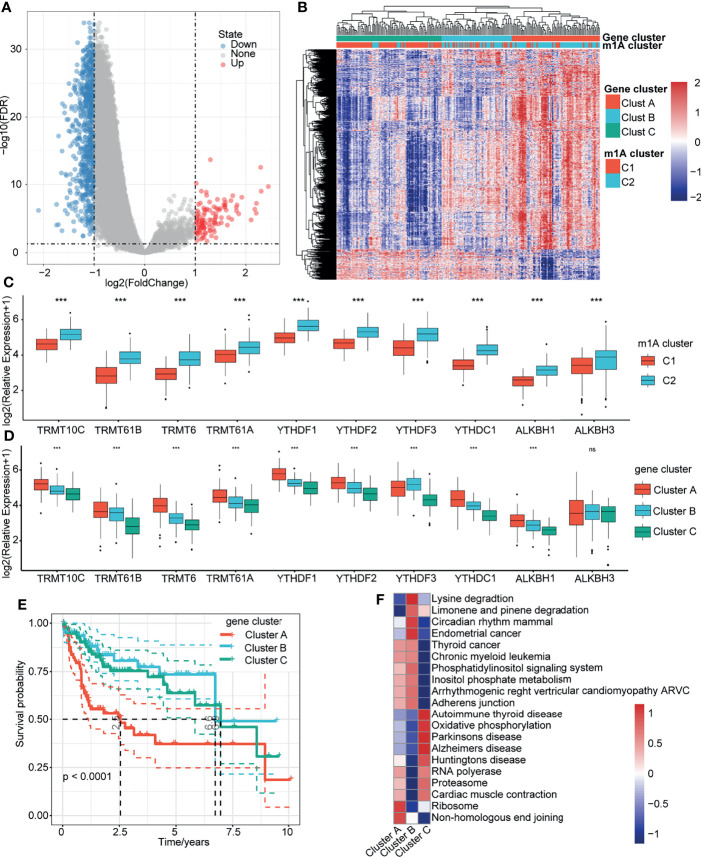
Gene clusters related to m1A modification patterns. **(A)** Volcano map of the DEGs in m1A Clusters C1 and C2. **(B)** Heatmap of m1A-related DEG unsupervised clustering. **(C)** Distribution of 10 m1A regulator genes in C1 and C2. **(D)** Distribution of 10 m1A regulator genes in gene clusters A, B, and C. **(E)** Overall survival differences among the three gene clusters. **(F)** Enrichment pathways of the three gene clusters. ***P < 0.001; ns, nonsense.

To determine which genes were associated with the m1A modification patterns, we used the limma package to identify the DEGs between m1A clusters C1 and C2. The volcano map of the DEGs is shown in **Figure 4A**. Then, we identified three gene clusters (clusters A, B and C) related to the DEGs through unsupervised cluster analysis (**Figure 4B**). We also found that C1 with a better prognosis was mainly distributed in Cluster C, while Cluster A contained the fewest samples of C1 (**Figure 4B**). Furthermore, we explored the expression of these 10 genes in the m1A clusters and m1A-related gene clusters (**Figures 4C, D**). Survival analysis showed that patients in Cluster A tended to have the worst prognosis, and those in Cluster C had the best prognosis (**Figure 4E**). Then, we performed GSVA on these three gene clusters by calculating the average enrichment score of the pathways in each gene cluster. The top 20 pathways with the largest differences were selected for visualization. The results showed that Cluster A was mainly enriched in nonhomologous end-joining and ribosomes, while Cluster B was mainly enriched in lysine degradation, limonene and pinene degradation, circadian rhythm mammals, and endometrial cancer. Cluster C was enriched in autoimmune thyroid disease, oxidative phosphorylation, Parkinson’s disease, Alzheimer’s disease, Huntington’s disease, RNA polymerase, proteasome, and cardiac muscle contraction (**Figure 4F**).

### Construction of a Prognostic Model Based on the DEGs Between m1A- Related Gene Clusters

**Figure 5:**
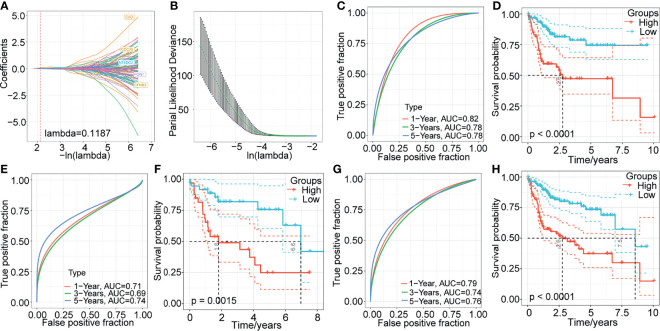
Construction and validation of a prognostic model based on the DEGs between m1A-related gene clusters. **(A)** The change trajectory of each independent variable. **(B)** The confidence interval under each lambda. **(C–H)** Time-dependent ROC curve measuring the predictive value of the five‐gene model in the TCGA training set, TCGA-LIHC validation dataset, and whole TCGA-LIHC dataset. Kaplan-Meier curves for overall survival by the 5-gene model in the TCGA training set, TCGA-LIHC validation dataset, and whole TCGA-LIHC dataset.

To identify potential prognostic biomarkers among the m1A-related genes, we constructed a prognostic risk model. First, we randomly divided 232 samples from the TCGA-LIHC dataset into a training set (n=162) and a validation set (n=70). The sample information is shown in **Table 2**. In the training set, 853 DEGs and survival data were analyzed, and 292 genes associated with HCC prognosis were identified by univariate Cox regression analysis. Then, LASSO Cox regression analysis was used to further narrow down the 292 DEGs. The change trajectory of each independent variable is shown in **Figure 5A**. With the gradual increase in lambda, the number of independent variable coefficients that tended to 0 increased (**Figure 5B**). The model reached the optimum when lambda = 0.1187. For this reason, we chose lambda = 0.1187, and finally, 5 genes were screened for further analysis. Then, we performed multivariate Cox regression analysis to establish the prognostic model. The risk score was calculated by summing the risk coefficient of each gene. The final risk score formula was as follows: 


Risk score=-0.048*PON1-0.105*CFHR3+0.290*CAD+0.053*NT5DC2+0.156*CDC20

**Table 2:** The sample information in training set and validation set.

Clinical Features	TCGA-train	TCGA-test	P
**OS**			
0	115	41	0.08966
1	47	29	
**T stage**			
T1	83	32	0.4374
T2	38	13	
T3	35	21	
T4	6	4	
**Stage**			
I	82	31	0.3966
II	37	13	
III	41	24	
IV	2	2	
**Grade**			
G1	20	9	0.5613
G2	69	32	
G3	64	28	
G4	9	1	
**Gender**			
Female	22	50	1
Male	48	112	
**Age**			
>60	39	89	1
<=60	31	73	

Next, we evaluated the sensitivity and specificity of the five-gene model using the area under the curve (AUC) of a time‐dependent receiver operating characteristic (ROC) curve (**Figure 5C**). The prognoses of the high- and low-risk groups were significantly different (P < 0.0001; **Figure 5D**). Additionally, to validate the stability of the five-gene-based model in predicting the overall survival of patients with HCC, we assessed the risk model in the TCGA-LIHC validation set and the whole TCGA-LIHC dataset. In TCGA-LIHC validation dataset, the AUCs at 1, 3, and 5 years were 0.71, 0.69, and 0.74, respectively (**Figure 5E**), and the high-risk group presented a significantly poorer prognosis than the low-risk group (**Figure 5F**). Similarly, the five-gene model also showed excellent predictive efficiency, and the prognosis was poorer for high-risk patients than for low-risk patients in the whole TCGA-LIHC dataset (**Figures 5G, H**).

### Generation of the m1A-Score Model

**Figure 6:**
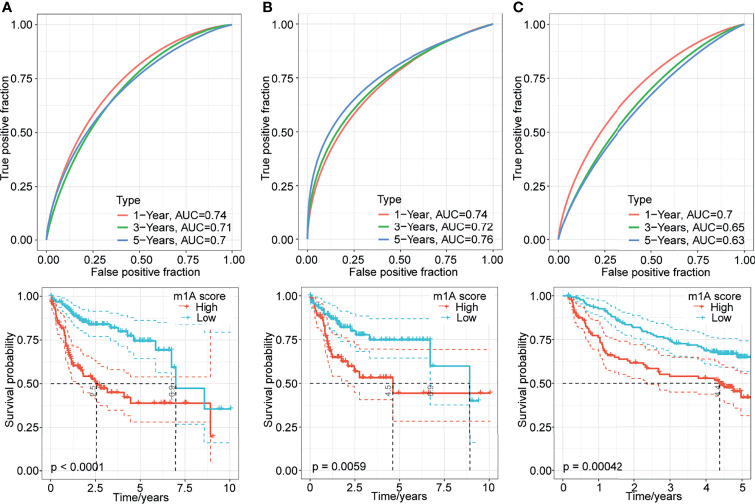
Generation of the m1A-score model. **(A–C)** Time-dependent ROC analysis and Kaplan-Meier analysis of the five-gene model in the TCGA-LIHC training dataset, entire TCGA-LIHC dataset, and GSE14520 dataset.

The above results suggested the impact of m1A-related genes on the prognosis of patients with HCC. To systematically analyse this impact on individuals, we established a scoring system named the m1A-score model. First, we performed principal component analysis to construct the m1A-score model. Principal components 1 and 2 were used to calculate the signature scores for each sample. The calculation formula was as follows: m1A score= ΣPC1i+PC2i where i represents the expression of 5 genes (PON1, CFHR3, CAD, NT5DC2 and CDC20). We assessed the m1A-score value of each sample based on the expression levels of these 5 genes in the sample. Then, we performed ROC analysis for the prognostic classification of the m1A score model (**Figure 6A**). The results of survival analysis showed that the prognosis of patients in the high- and low-risk groups was different (**Figure 6A**). To further determine the robustness of this model, the TCGA training dataset and GSE14520 dataset were analyzed. Consistent with the results of the TCGA-LIHC training dataset, patients with high m1A scores tended to have poorer survival than patients with low m1A scores, and the AUC values were all above 0.6 for the entire TCGA-LIHC dataset (**Figure 6B**) and the GSE14520 dataset (**Figure 6C**). The evidence collectively suggested that the m1A-score model may be a stable scoring tool for predicting the survival of HCC patients.

### Correlation Analysis of Immune Regulation and the m1A Score

**Figure 7:**
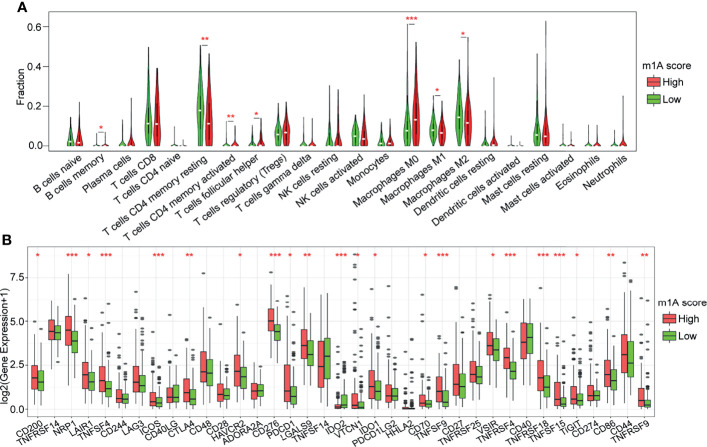
Correlation analysis of immune regulation and the m1A score. **(A)** Distribution of 22 types of immune cells in the high- and low-m1A score groups. **(B)** Expression of 37 immune checkpoints in the high- and low-m1A score groups. *P < 0.05, **P < 0.01, ***P < 0.001.

To further investigate the association between the m1A score value and the TME of HCC, we evaluated the levels of immune cell infiltration and the expression of immune checkpoint genes in distinct m1A score groups. We found that approximately 31.82% of the 22 immune cells were significantly different between the two groups using the CIBERSORT method (**Figure 7A**). Then, the expression of 37 immune checkpoints published in a previous study (20) was assessed in the two groups (**Figure 7B**). The results showed that approximately 56.76% (21) of the 37 immune checkpoints had significant differences. For example, the high-m1A score group showed higher expression levels of CD200, NRP1, LAIR1, TNFSF4, ICOS CTLA4, HAVCR2, CD276, PDCD1, LGALS9, IDO1, CD70, TNFSF9, TNFRSF9, TNFSF18, TNFSF15, CD86, and CD44 (**Figure 7B**), whereas the lowm1A score group was more correlated with high expression of IDO2. The results above indicate that the patients in the high-m1A score group may respond poorly to immune checkpoint drugs, which needs to be further researched.

### Predictive Performance of the m1A-Score Model in Clinical Application

After confirming the correlation of the m1A score and TME cell infiltration characteristics, it was subsequently investigated whether the scoring model could be applied to predict the prognosis of patients with different clinical features. We found that the m1A score was markedly correlated with prognosis in patients older than or younger than 60 years of age, of different sexes, and with different TNM stages, grades and stage statuses (**Figure S3**, all P<0.01).

### The m1A-Score Model Might Serve as an Independent Prognostic Biomarker in Clinical Application

**Figure 8:**
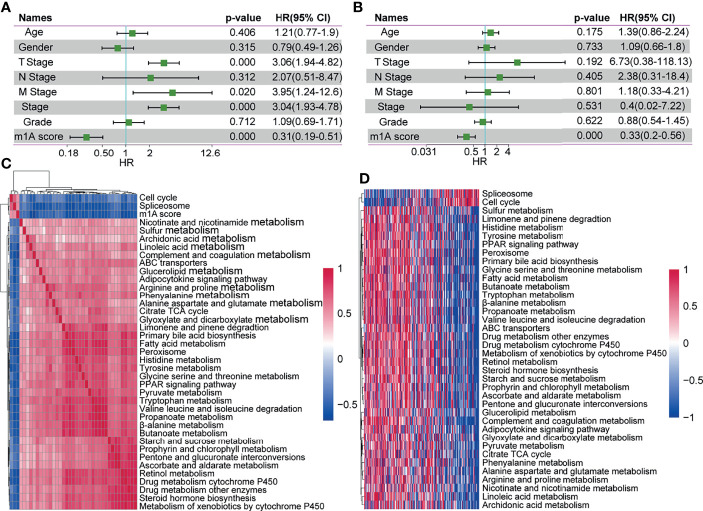
The m1A-score model might serve as an independent prognostic biomarker in clinical application. **(A, B)** Univariate regression analysis and multivariate regression analysis of the m1A score in the whole TCGA-LIHC cohort. **(C)** Clustering of correlation coefficients between KEGG pathways with a risk score correlation greater than 0.4. **(D)** KEGG pathway analysis with a risk score correlation greater than 0.4. As the m1A score increases, the ssGSEA in each sample changes in score.

To identify whether the m1A-score model could serve as an independent biomarker for prognosis, we performed univariate and multivariate Cox regression analyses of clinical data to assess the relevant hazard ratios (HRs) and 95% confidence intervals (CIs) in the entire TCGA-LIHC dataset. The results of univariate regression analysis revealed that the m1A score was significantly related to survival (**Figure 8A**). Multivariate Cox regression analysis showed that the m1A score was an independent risk factor for prognosis (**Figure 8B**). The above findings showed that the m1A-score model has good predictive performance for the prognosis of patients in clinical application.

### Association Between the m1A Score and KEGG Pathways

To explore the correlation between biological behaviours and different m1A scores, we performed functional annotations of TCGA-LIHC samples using ssGSEA *via* the GSVA R package. After we obtained the ssGSEA score for different functions in each sample, we further explored the correlation between these functions and the m1A score (correlation coefficient >=0.4). Finally, a total of 36 KEGG pathways were negatively correlated with the sample m1A scores (for example, peroxisome), and 2 pathways (including cell cycle and spliceosome) were positively correlated with the sample m1A scores (**Figure 8C**). Cluster analysis based on the 36 KEGG pathways was performed according to their enrichment scores. We found that the pathways of the cell cycle and spliceosome increased with increasing m1A scores (**Figure 8D**).

## Discussion

m1A regulators govern m1A RNA methylation functions. Some research groups have reported that m1A regulators play important roles in the progression of tumorigenesis. In this study, we described the genetic variations in m1A regulators in HCC and found that the changes in m1A regulators were correlated with the prognosis of HCC patients. Similarly, Shi et al. (18) observed a high mutation frequency in the 10 m1A regulators using TCGA-LIHC dataset and identified four regulators that were significantly correlated with prognosis. In ovarian cancer, Liu et al. (21) found that three different m1A modification patterns which could predict patient survival, stage and grade. In pancreatic cancer, m1A regulator genetic variations are related to clinical stage, and CNVs are closely associated with the expression of m1A regulators. Notably, the expression level of ALKBH1 is closely associated with the prognosis of patients with pancreatic cancer (22). Li et al. (23) systematically analyzed the association of the molecular alterations of m1A regulators and the clinical data of 33 cancer types from the TCGA. This group found that m1A regulatory protein expression was correlated with various carcinogenic pathways and patient overall survival, indicating that m1A regulators have the potential for prognostic prediction in many types of cancer and may now provide new treatment strategies (23).

Increasing evidence has revealed that RNA methylation modifications influence the formation of the TME and the immune cell-infiltrating characteristics of the tumor; thus, the association between the RNA modification represented by m6A and the TME has aroused extensive interest from researchers (24). Zhang et al. (25) identified three m6A modification patterns and found that the immune cell-infiltrating features under these three patterns were highly consistent with the well-known immune phenotypes, namely, the immune-inflamed, immune-desert and immune-excluded phenotypes. In colon cancer, Chong et al. (26) also identified three m6A modification patterns, and those patterns were highly consistent with the three immune phenotypes, suggesting that m6A was correlated with the diversity and complexity of the TME. Yi et al. (27) reported that m6A regulators were significantly correlated with PD-L1 expression and distinct immune cell infiltration in head and neck squamous cell carcinomas. The association between m6A modification patterns and the TME was also assessed in lung adenocarcinoma and gliomas (28, 29). Nevertheless, the role of m1A in the TME is still unclear. Here, we identified 2 distinct m1A modification patterns (Clusters 1 and 2) based on the expression of 10 m1A regulators. We extracted clinical information from TCGA-LIHC and found that patients in Cluster 1 had longer overall survival times than those in Cluster 2. We also compared the immune cell infiltration characteristics of different m1A modification patterns using the CIBERSORT method. After construction of the m1A-score model, we evaluated the immune cell infiltration levels and expression of immune checkpoint genes in distinct m1A score groups. We found that several of the 22 immune cells were significantly different between the two groups. In addition, the expression of 37 immune checkpoints was significantly different between the two groups. These results might enhance our understanding of the function of m1A modification in the formation of a complex TME in HCC.

Here, we not only identified the m1A modification patterns of HCC samples from the TCGA and GEO databases but also constructed the m1A-score model and systematically analyzed its impacts on individuals. The results of ROC and survival analyses indicated that the m1A-score model could serve as a stable scoring tool for predicting the survival of HCC patients. Additionally, analysis of the clinical information of the entire TCGA-LIHC dataset to determine the HR showed that the m1A-score model was an independent biomarker for prognosis in clinical application. Conclusively, evaluating the m1A modification patterns of individual patients with HCC will enhance our understanding of the characteristics of TME infiltration and provide novel ideas for prognostic biomarkers and therapeutic strategies.

## Conclusion

We identified two distinct m1A modification patterns which were associated with different overall survival and TME characteristics of patients with HCC. In addition, we constructed an m1A-score model to quantitatively evaluate the m1A modification patterns of individual patients which might be served as effective biomarkers for predicting the prognosis of patients with HCC.

## Data Availability Statement

The original contributions presented in the study are included in the article/**Supplementary Material**. Further inquiries can be directed to the corresponding authors.

## Author Contributions

CX conceived and planned the study design. SS and MZ performed formal analysis and data interpretation. MZ wrote the original draft. SS provided critical revisions and contributed to the editing of the paper. All authors read and approved the final manuscript.

## Funding

This work was supported by grants from the Science and Technology Research Project of Henan Province (202102310115), and Henan Medical Science and Technology Joint Building Program (LHGJ20200387).

## Conflict of Interest

The authors declare that the research was conducted in the absence of any commercial or financial relationships that could be construed as a potential conflict of interest.

The reviewer W.G. declared a shared affiliation with the authors to the handling editor at time of review.

## Publisher’s Note

All claims expressed in this article are solely those of the authors and do not necessarily represent those of their affiliated organizations, or those of the publisher, the editors and the reviewers. Any product that may be evaluated in this article, or claim that may be made by its manufacturer, is not guaranteed or endorsed by the publisher.

## Supplementary Material

Supplementary Figure 1 Features of genetic variations in m1A regulators in HCC. **(A)** Correlation between the expression of each regulator and CNVs. **(B)** SNV mutation of m1A regulators.

Supplementary Figure 2 GSVA of m1A-related patterns.

Supplementary Figure 3 The m1A-score model was applied to predict the prognosis of HCC patients with different clinical characteristics.
