# Supplementary figures and images for: A Novel m1A-Score Model Correlated With the Immune Microenvironment Predicts Prognosis in Hepatocellular Carcinoma
Source: Front Immunol. 2022 Mar 24;13:805967. doi: 10.3389/fimmu.2022.805967 (PMC8987777; doi:10.3389/fimmu.2022.805967)

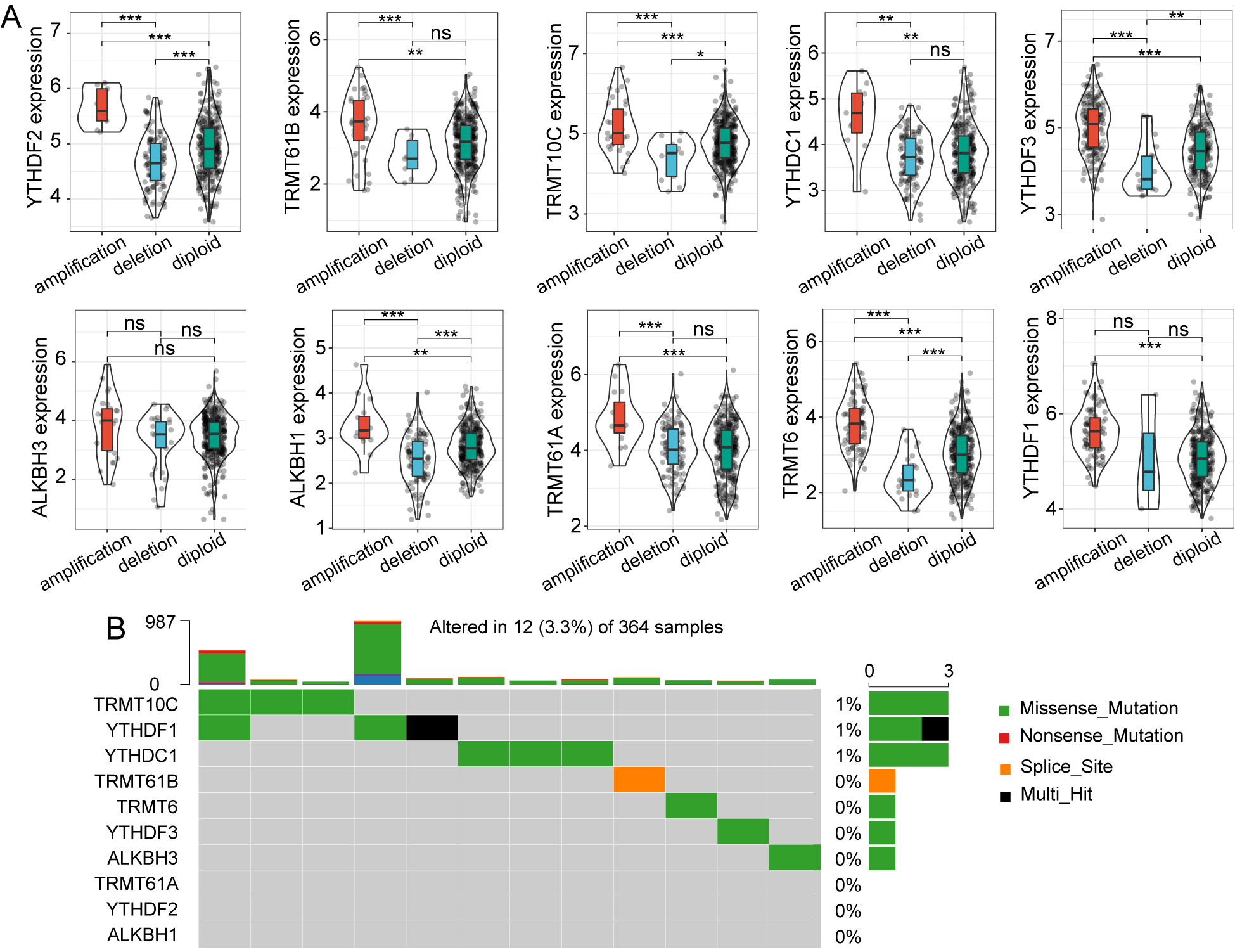

Supplement: Supplementary Figure 1 — Features of genetic variations in m1A regulators in HCC. (A) Correlation between the expression of each regulator and CNVs. (B) SNV mutation of m1A regulators. [file Image_1.tif]

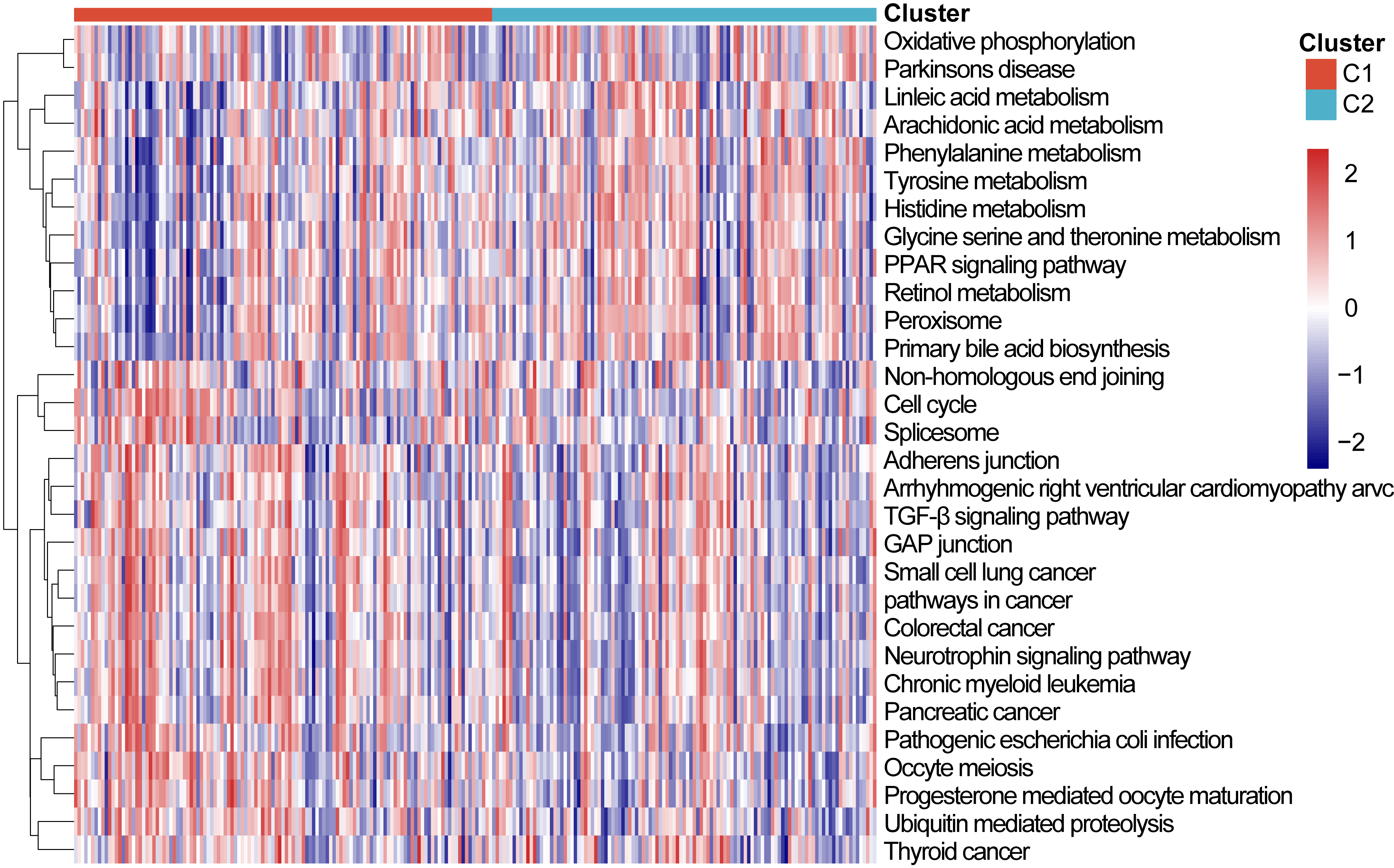

Supplement: Supplementary Figure 2 — GSVA of m1A-related patterns. [file Image_2.tif]

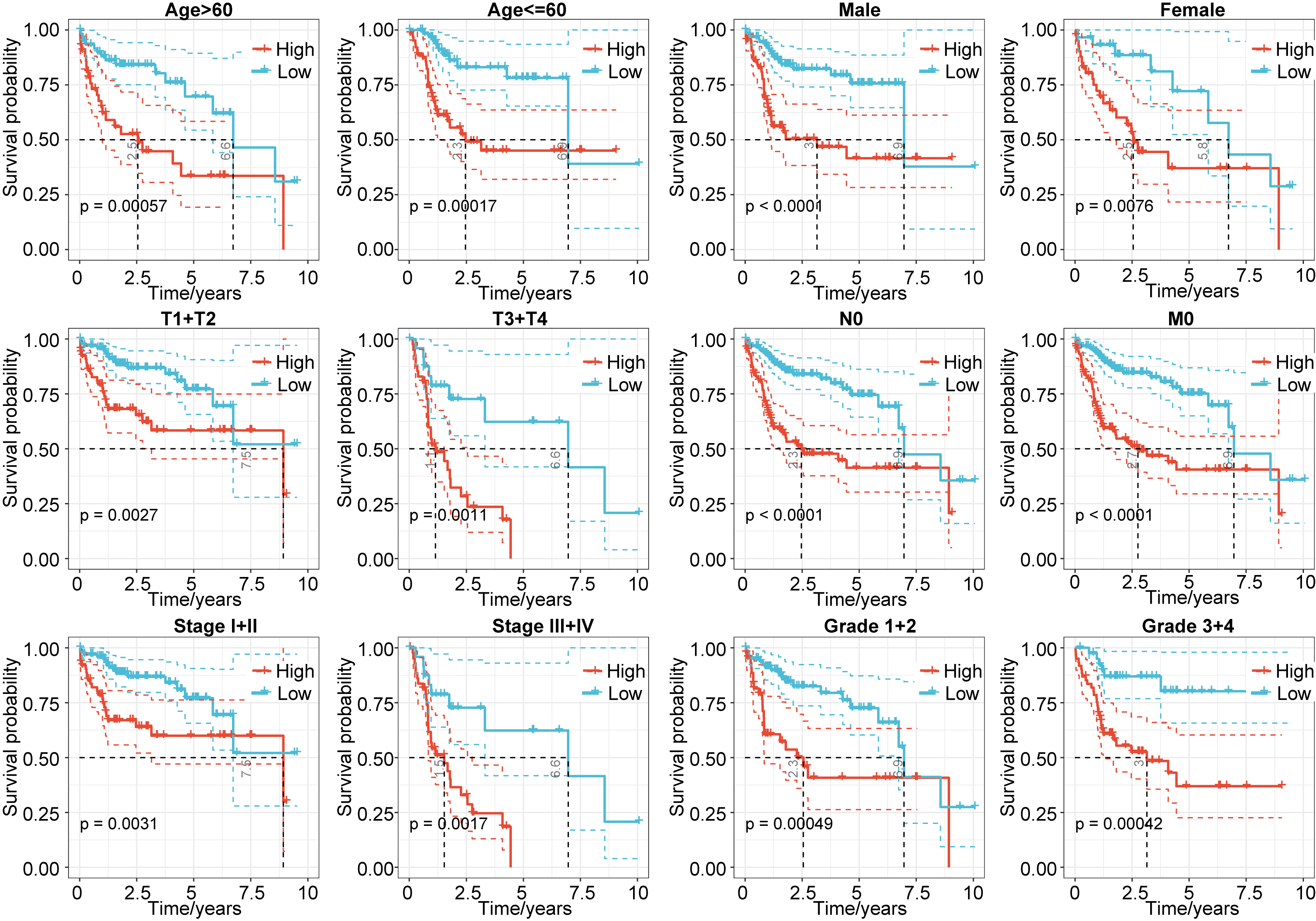

Supplement: Supplementary Figure 3 — The m1A-score model was applied to predict the prognosis of HCC patients with different clinical characteristics. [file Image_3.tif]
